# Complementary Radiographic Projection for Evaluation of the Conchal Sinuses and Bullae in Horses

**DOI:** 10.1111/vru.70046

**Published:** 2025-05-15

**Authors:** Rubens Peres Mendes, Aymara Eduarda de Lima, Reginaldo da Cunha, Mauricio Jose Bittar, Christian Carlstron Vasconcelos, Diego Darley Velasquez Piñeros, Rodrigo Romero Corrêa

**Affiliations:** ^1^ Department of Surgery, School of Veterinary Medicine and Animal Science University of São Paulo São Paulo Brazil; ^2^ Self‐Employed Veterinarian, Horse Dentist Brasil São Paulo Brazil; ^3^ Self‐Employed Veterinarian Jaguariúna Brazil; ^4^ Self‐Employed Veterinarian, BittarVet Rio Claro Brazil; ^5^ Self‐Employed Veterinarian, Sed Bogotá Colombia

**Keywords:** radiography, paranasal sinuses, nasal conchae, sinusitis, empyema

## Abstract

Radiographic examination of the skull is a well‐established and indispensable procedure for the diagnostic evaluation of dental and paranasal sinus disorders in horses. Complementary projections make significant contributions to radiographic diagnosis in nasal conchae disorders. This article describes a complementary radiographic projection designed for the evaluation of the conchal sinuses and bullae in horses. Six equine cadaveric heads were used. Specimens were dissected, and openings were created in the ventral and dorsal nasal conchae for the introduction of radiopaque material. The new radiographic projection was named lateral 75° dorsal–lateral ventral oblique view. This projection was obtained with the mandibular rami resting on the imaging plate and the mandible displaced toward the side of interest (partial excursion). The X‐ray beam was directed dorsoventrally, slightly angled toward the side of interest (left or right offset mandible dorsoventral view), and centered at an imaginary line connecting the tips of the facial crests. The lateral 75° dorsal–lateral ventral oblique view provided enhanced visualization and allowed correct identification of equine conchal structures. Lateral displacement of the mandible and the 75° angle of inclination to the vertical plane eliminated the superimposition of anatomical structures, facilitating radiographic image interpretation and increasing diagnostic accuracy. This complementary projection is recommended in all cases of sinonasal disorders in horses.

## 1 Introduction

The dorsal and ventral nasal conchae and paranasal sinuses form the complex equine paranasal system [1]. They are divided into a caudal and a rostral portion by a transverse septum. The caudal portions are named ventral (VCS) and dorsal (DCS) conchal sinuses, while the rostral portions are described as ventral (VCB) and dorsal (DCB) conchal bullae (International Committee on Veterinary Gross Anatomical Nomenclature, [2]).

Sinonasal diseases are the most common cause of unilateral purulent nasal discharge in horses [3]. Empyema of the conchal bullae is an important cause of chronic nasal discharge. In horses with paranasal diseases diagnosed by CT, 23% of the conchal bullae were affected [4]. The VCS is also prone to pus accumulation [5]. This compartment was found to be the second most affected in sinus disorders (261 out of 300 cases; 87 %) [6].

CT is the gold standard for the diagnosis of sinus disorders [4, 7, 8]. However, since CT is still limited to referral centers, radiography remains a valuable diagnostic imaging modality in horses [8].

Radiographic examination of the skull is a well‐established procedure [9] and is fundamental for the diagnostic assessment of cheek teeth and paranasal sinuses [10]. This imaging modality is widely used in horses presenting with rhinorrhea, unilateral foul‐smelling nasal discharge, epistaxis, or facial asymmetries [9].

Standard radiographic projections used to evaluate the nasal cavity and paranasal sinuses include the right and left laterolateral, latero‐30° dorsal–lateroventral oblique, and dorsoventral views [8, 11]. Although the VCB and DCB can be identified using these projections [6], special projections, such as the offset mandible dorsoventral view, may increase diagnostic accuracy in cases of sinonasal abnormalities involving the conchal sinuses and bullae [12].

Complementary projections are recommended for comprehensive radiographic investigation and control studies [7]. This study set out to describe a complementary radiographic projection for the evaluation of the conchal sinuses and bullae in horses. The new projection was based on the radiographic identification of sinonasal structures on the dorsoventral view [6] and on anatomical and radiographic descriptions of the conchal sinuses and bullae on the offset mandible dorsoventral view [10].

## 2 Materials and Methods

### 2.1 Specimens

The study was approved by the Ethics Committee on Animal Use of the institution. The complementary projection described in this study was developed and standardized at the Equine Dentistry Center (COE) of the School of Veterinary Medicine and Animal Science, University of São Paulo (FMVZ‐USP), Pirassununga—SP. Six cadaveric heads of client‐owner horses who died or were euthanized for reasons unrelated to the study and had no clinical history of sinus or dental disorders were included in this study. Donor animals were aged eight to 25 years (mean age, 14.5 years). Specimens were chemically preserved as per Corrêa et al. [13], and stored at 2°C to 6°C until use.

### 2.2 Radiographic evaluation

Radiographic evaluations were previously performed using laterolateral, dorsoventral, left and right lateral 30° dorsal–lateroventral oblique and dorsoventral offset views and radiographic changes consistent with sinus abnormalities used as exclusion criteria.

Specimens were radiographed using the complementary projection before and after the introduction of radiopaque material. Radiographic images were obtained using the following settings: 80 kV, 3.2 mAs (20 mA), and 75 cm film‐focus distance (FFD). The X‐ray device used was a portable DR system digital detector (ABLA Import) with a digital generator (Poskom).

**FIGURE 1:**
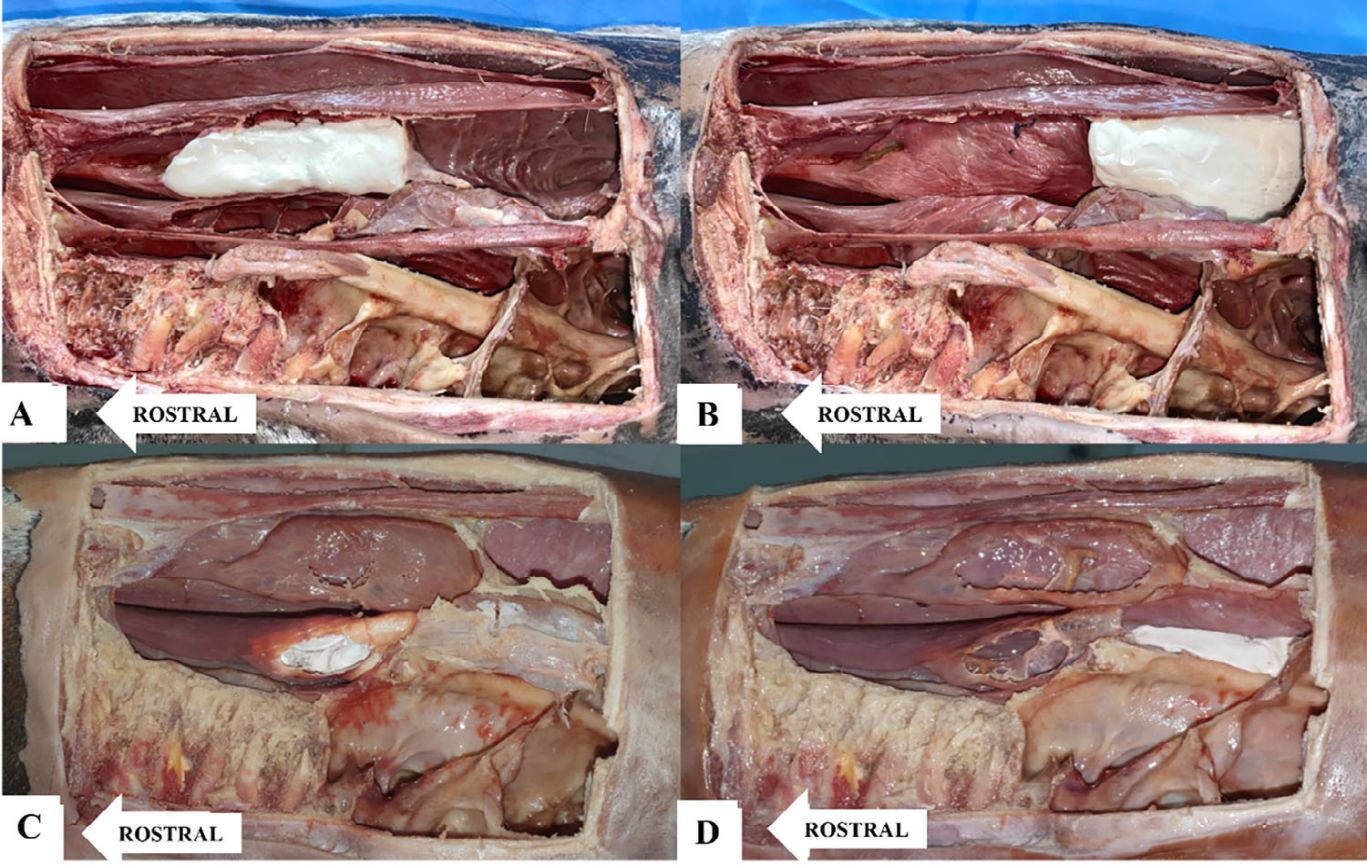
Lateral view of conchal bullae and sinuses packed with radiopaque material. A, Dorsal conchal sinus. B, Dorsal conchal bulla. C, Ventral conchal sinus. D, Ventral conchal bulla.

A bone flap was created. Soft tissues overlying a rectangular area of the maxillary region were dissected away to expose the bony surface. This area was demarcated by the medial corner of the eye, the nasal bone (1 cm lateral to the sagittal plane), the nasoincisive notch, and the facial crest (caudal, dorsal, rostral, and ventral limits respectively) (Figure 1).

Lateral conchotomy was performed in the dorsal conchal sinus (DCS), the dorsal conchal bullae (DCB), and the ventral conchal sinus (VCS) on both sides.

A small incision was made using scissors, through which barium sulfate‐based radiopaque contrast agent (BarioGel 100%) was applied with a brush to the lateral, caudal, rostral, medial, dorsal, and ventral walls of the VCB until complete coverage was achieved. The DCB, DCS, and VCS were filled with condensation silicone (Perfil Kit). Each structure was marked individually, one side at a time.

Radiographs were taken of the marked structures on one side, after which the radiopaque material was carefully removed using water‐soaked gauze to avoid damaging the anatomical features. The contrast agent was then applied to the contralateral side, and a new set of radiographs was obtained. Photographic documentation was carried out before and after filling the structures.

### 2.3 Lateral 75° Dorsal–Lateral Ventral Oblique View: Radiographic Technique

To acquire the novel lateral 75° dorsal–lateral ventral oblique projection, the radiographic detector was positioned ventral to the horizontal rami of the mandible, with its rostral edge aligned with either the interdental space or the incisor teeth. The angle of the X‐ray beam was determined using a universal angle protractor by establishing two reference lines: one perpendicular and sagittal to the frontal bone of the specimen and a second representing the beam entry angle.

The mandible was displaced toward the side of interest (partial lateral excursion) using a radiographic mouth speculum (Pro Horse Dental). The extent of displacement corresponded to one‐third of the speculum's maximum opening, approximately the width of a single incisor. This adjustment resulted in the superimposition of the upper and lower cheek teeth on the side of interest.

**FIGURE 2:**
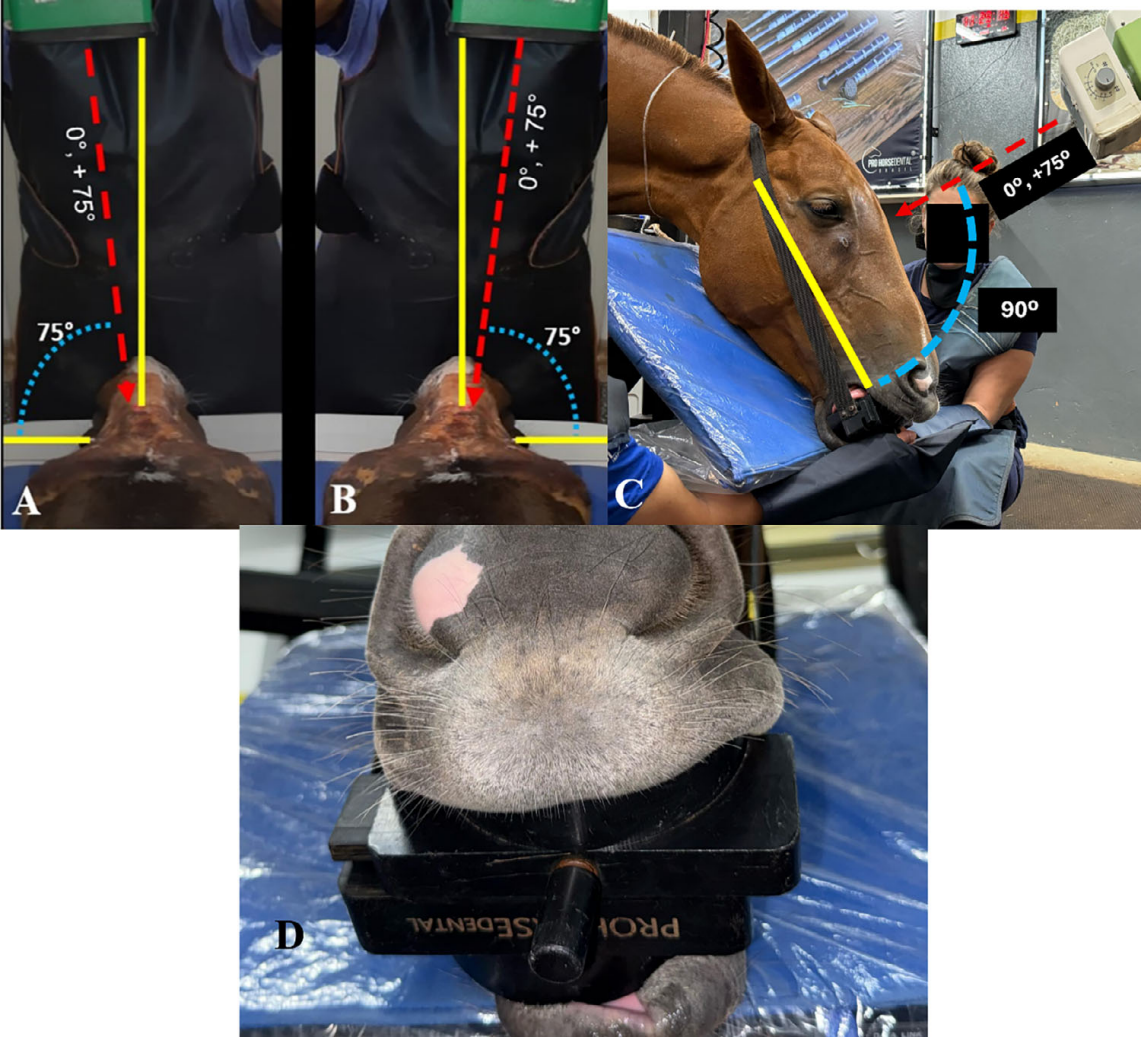
Complementary lateral 75° dorsal–lateral ventral oblique. Caudal view (A). X‐ray beam at a 75° angle to the left, combined with dislocation of the mandible to the same side. Dorsal view (B). X‐ray beam at a 75° angle to the right, combined with dislocation of the mandible to the same side. Lateral view (C). Position of the X‐ray beam relative to the imaging plate. Frontal view (D). A radiographic mouth speculum (Pro Horse Dental) for displacement toward the side of interest (partial lateral excursion). Image for illustrative purposes only.

The X‐ray tube was positioned at a source‐to‐image distance of 75 cm, and the beam was directed dorsoventrally at a slight lateral angle toward the side being examined. The beam was centered along an imaginary line connecting the apices of the facial crests (Figure 2).

Radiographic images were evaluated based on the following criteria: clarity of anatomical structure visualization, degree of overlap reduction, absence of image distortion, and technical reproducibility. Image assessments were independently performed by four blinded reviewers, all of whom were experienced surgeons familiar with equine head radiography and paranasal sinus surgery.

## 3 Results

The new complementary projection allowed accurate identification of the dorsal and ventral conchal sinuses and bullae. Plain radiographs were used for the identification of the conchal sinuses and bullae, whereas contrast‐enhanced images provided detailed information about the location and compartmentalization of these structures.

**FIGURE 3:**
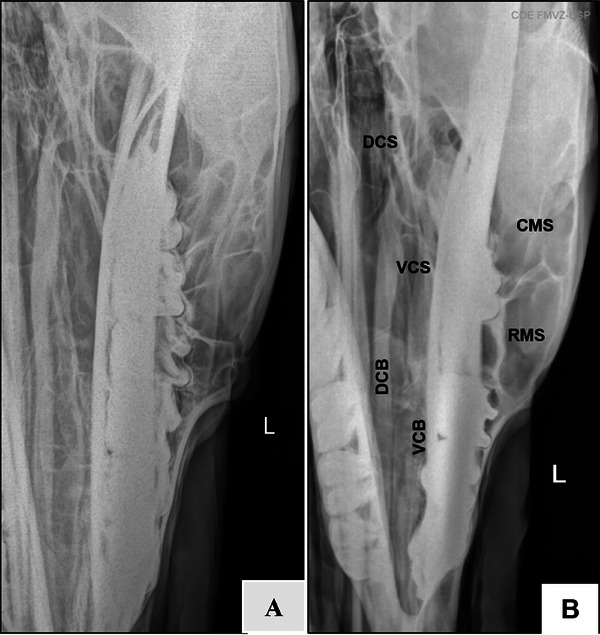
Radiographic image obtained using the complementary lateral 75° dorsal–lateral ventral oblique (A). Left side. Note overlapping mandibular and maxillary teeth and clearly visible ventral and dorsal nasal conchae (B). Left side. Note the nasal septum in contact with the fourth premolar and overlapping mandible and maxilla, with comprehensive visualization of the ventral and dorsal nasal conchae. DCS—dorsal conchal sinus; VCS, ventral conchal sinus; DCB, dorsal conchal bulla; VCB, ventral conchal bulla; CMS—caudal maxillary sinus; RMS, rostral maxillary sinus.

Lateral displacement of the mandible prevented overlap between the nasal conchae and mandibular teeth and allowed comprehensive visualization of the dorsal and ventral nasal conchae (Figure 3A). There was a slight overlap between the lateral aspect of the dorsal nasal concha and the medial aspect of the ventral nasal conchae. The nasal septum is in an oblique position, aligned with the contralateral mandibular fourth premolar (Triadan 08) (Figure 3B).

**FIGURE 4:**
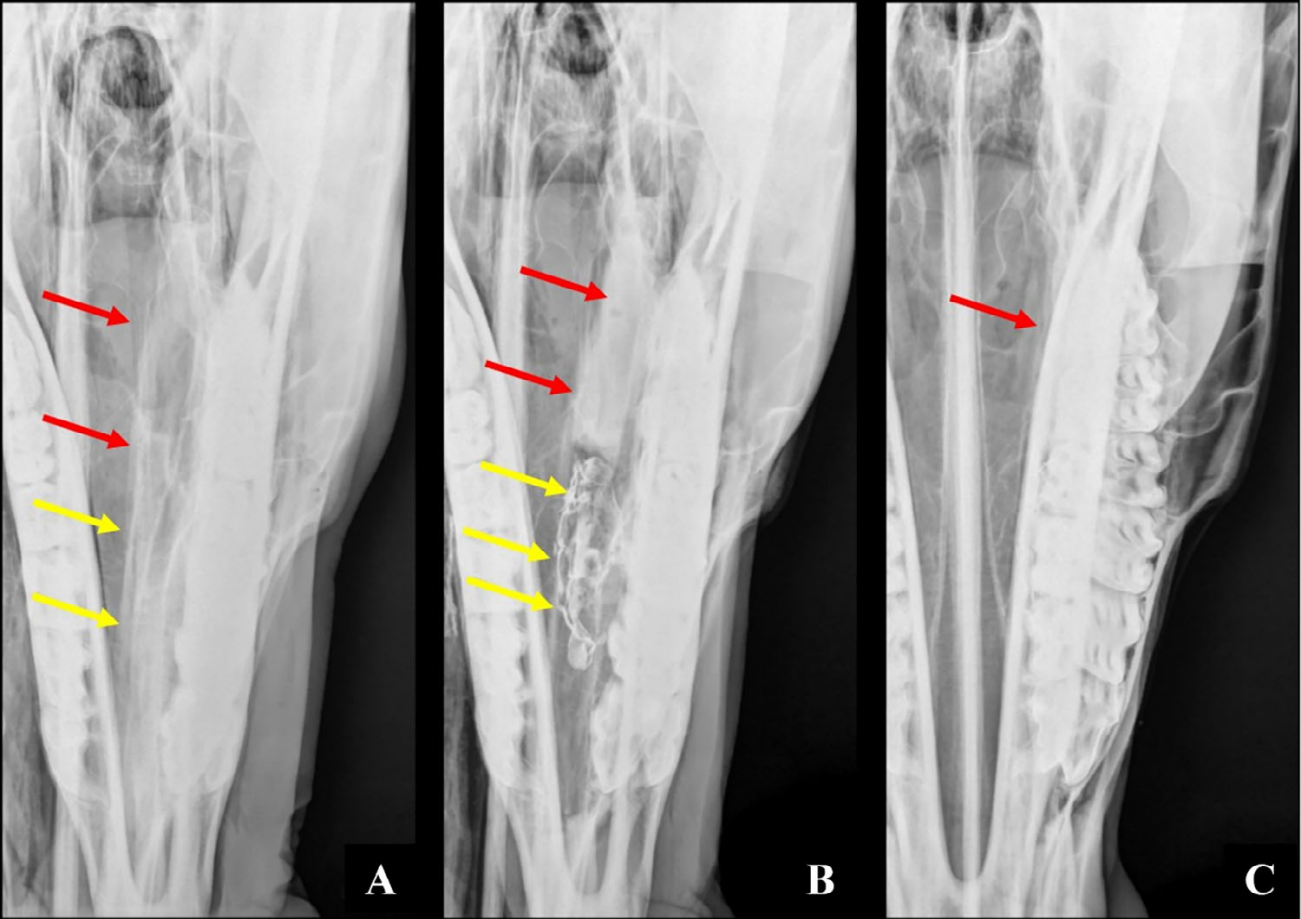
A, Left side. Contrast‐enhanced dorsoventral radiograph. The ventral concha is superimposed on the mandibular teeth (red arrow). B, Left side. Contrast‐enhanced complementary lateral 75° dorsal–lateral ventral oblique radiograph. Ventral conchal sinus (red arrows). Ventral conchal bulla (yellow arrows). C, Left side. Plain complementary lateral 75° dorsal–lateral ventral oblique radiograph. Ventral conchal sinus (red arrows). Ventral conchal bulla (yellow arrows).

The caudal limit of the VCS is at the level of the distal aspect of the maxillary third molar (Triadan 11), medial to the infraorbital canal, while the rostral limit was located at the level of the middle third of the maxillary first molar (Triadan 09). The mesial aspect of the third premolar (Triadan 07) formed the rostral limit of the VCB. The medial wall of the ventral nasal concha was separated from and parallel to the nasal septum, and the lateral wall was adjacent to the ipsilateral hemiarch (Figure 4).

The dorsal nasal concha is next to the nasal septum, away from the ipsilateral hemiarch. The DCS was bounded caudally by the rostral aspect of the Vomer bone, distal to the third maxillary molar (Triadan 11), and rostrally by the distal aspect of the second maxillary molar (Triadan 10). The ethmoid turbinates overlapped with the caudal portion of the DCS.

**FIGURE 5:**
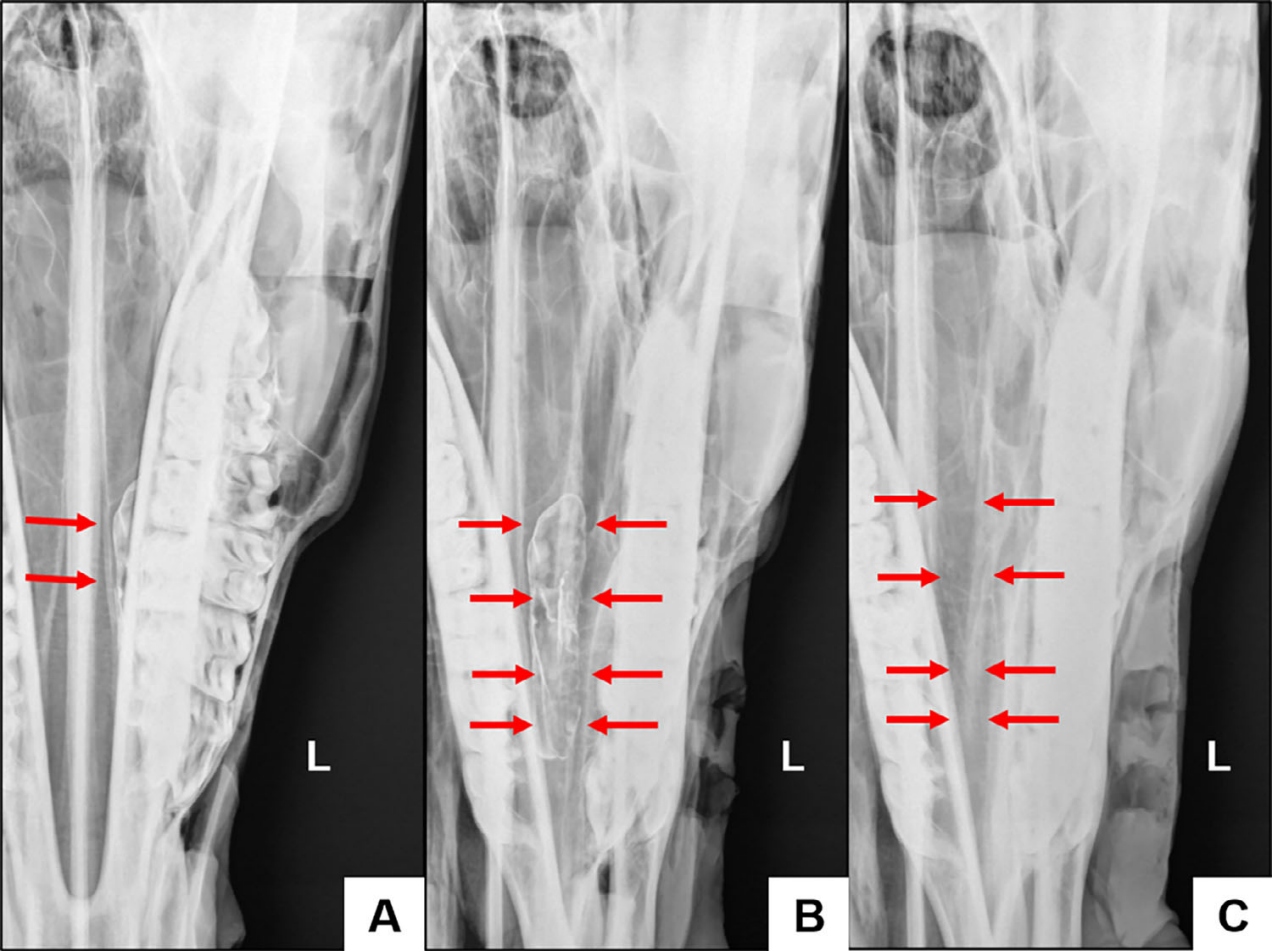
A, Left side. Contrast‐enhanced dorsoventral radiograph. The dorsal concha is superimposed on the mandibular teeth (red arrow). B, Left side. Contrast‐enhanced complementary lateral 75° dorsal–lateral ventral oblique radiograph. Dorsal conchal bulla (red arrows). C, Left side. Plain complementary lateral 75° dorsal–lateral ventral oblique radiograph. Dorsal conchal bulla (red arrows).

**FIGURE 6:**
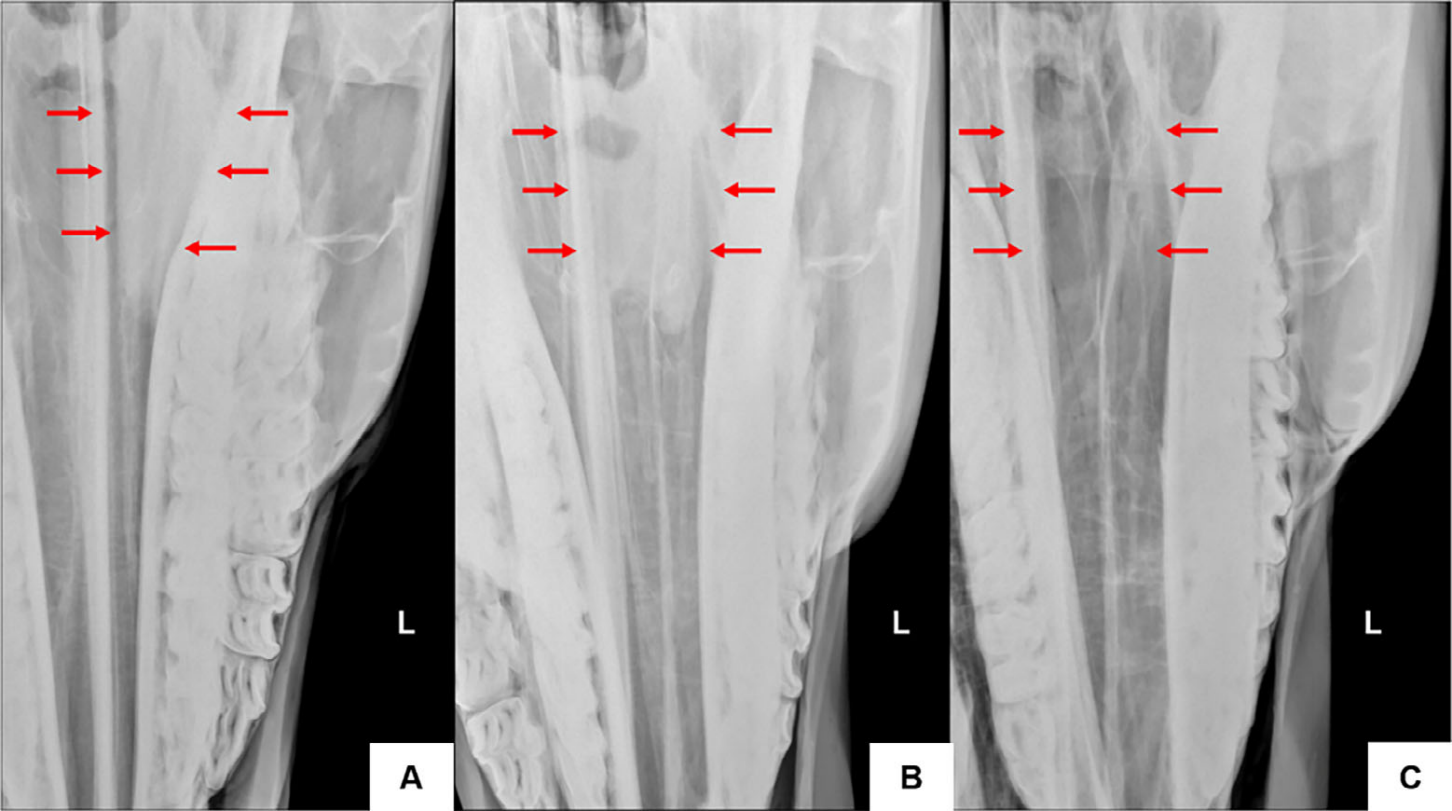
A, Left side. Contrast‐enhanced dorsoventral radiograph. The dorsal concha is superimposed on the mandibular teeth (red arrow). B, Left side. Contrast‐enhanced complementary lateral 75° dorsal–lateral ventral oblique radiograph. Dorsal conchal sinus (red arrows). C, Left side. Plain complementary lateral 75° dorsal–lateral ventral oblique radiograph. Dorsal conchal sinus (red arrows).

Small transverse septations were evident in the DCB, dividing it into different compartments (cells). The most caudal septation separates the DCB from the DCS. The mesial aspect of the mandibular third premolar (Triadan 07) formed the rostral limit of the DCB. The medial wall was parallel to the nasal septum up to the level of the fourth premolar, then continued along the medial lamina of the mandible. The lateral wall was parallel to the ipsilateral hemiarch (Figures 5 and 6).

## 4 Discussion

One of the biggest challenges in the interpretation of equine skull radiographs is the complexity of the radiographic image due to superimposition [7]. Images obtained using the lateral 75° dorsal–lateral ventral oblique projection prevented the overlap of cheek teeth, mandibular rami, and contralateral structures, and allowed accurate identification of the ventral and dorsal nasal conchae. However, given the two‐dimensional nature of the radiographic images and the complexity and proximity of the anatomical structures, overlap should be expected.

The lateral 75° dorsal–lateral ventral oblique view has not been described in the literature to date. The laterolateral, lateral 30° dorso‐lateroventral oblique, and dorsoventral views are the standard projections used for the diagnosis of sinus disorders in horses [6, 9]. Special radiographic projections, such as the offset mandible dorsoventral view, are recommended for improved diagnostic accuracy in suspected cases of sinonasal disorders [10].

The offset dorsoventral projection is primarily indicated for dental assessment [9]. However, since in this view, the mandible and nasal conchae do not overlap, it can also be used to evaluate the nasal conchae [10].

The complementary lateral 75° dorsal–lateral ventral oblique projection was developed according to the jaw displacement principle. Partial displacement of the mandible to the side of interest results in occlusal contact between the mandibular and maxillary teeth, avoiding cheek teeth and nasal conchae overlap and providing enhanced visualization of conchal structures.

The +75° angle of inclination of the X‐ray beam relative to the vertical plane facilitates the visualization of the nasal conchae and prevents the superimposition of the medial lamina of the mandible on the lateral conchal wall. Conchal structures can also be clearly seen since the dorsal and ventral conchae do not overlap. The overlap between the lateral aspect of the dorsal nasal concha and the medial aspect of the ventral nasal concha may be due to the close anatomical relationship between these structures.

In the standard dorsoventral projection, the VCB is superimposed on the mandibular teeth, while the lateral and rostral portions of the DCB are partially hidden by the mandible [6, 10].

Correct identification of the VCS on dorsoventral radiographs has been described [9]. However, according to Piñeros et al. [10], the VCS and the mandibular second and third molars overlap in this view. In the complementary projection, the VCS and the VCB are clearly visible between the nasal septum and ipsilateral hemiarches without superimposition. Hence, this radiographic view is highly recommended in suspected cases of VCS disease.

Overlapping of the rostral and caudal maxillary sinuses with dental apices in young horses limits the assessment of the ventral conchal bulla on laterolateral and lateral 30° dorso‐lateroventral oblique views [9, 14]. The use of specimens obtained from horses aged 14.5 years on average ensured the nasal conchae were fully developed so that maxillary dental apices would not interfere with the radiographic identification of the conchal bullae and sinuses.

The radiographic settings employed provide appropriate contrast between conchal structures. In some images, the bony septations separating the conchal sinuses and bullae can also be seen. The radiographic exposure technique may differ due to anatomical differences in skull shape [7] and overlapping molar dental apices in young horses [12]. CT allows accurate identification of the nasal conchae [12]. However, anatomical and radiographic descriptions of the conchal sinuses and bullae provided by Giavitto and Barakzai [6] and Piñeros et al. [10] support the radiographic assessment of these structures and underscore the role of radiography as an important imaging modality in the diagnosis of equine sinonasal disease [6].

## 5 Conclusion

The lateral 75° dorsal–lateral ventral oblique projection provides enhanced visualization and allows correct identification of equine conchal structures. Lateral displacement of the mandible and the +75° angle of inclination to the vertical plane prevent the overlapping of nasal conchae and adjacent structures. Radiographic image interpretation is thus facilitated and aids diagnostic evaluation. This complementary projection is recommended in all cases of sinonasal disorders in horses.

## List of Author Contributions

### Category 1

(a) Conception and design: Mendes, da Cunha, Vasconcelos
(b) Acquisition of data: Mendes
(c) Analysis and interpretation of data: Mendes, de Lima, da Cunha, Bittar, Vasconcelos, Piñeros, Corrêa

### Category 2

(a) Drafting the article: Mendes
(b) Revising article for intellectual content: Mendes, de Lima, da Cunha, Bittar, Vasconcelos, Piñeros, Corrêa

### Category 3

Final approval of the completed article: Mendes, de Lima, da Cunha, Bittar, Vasconcelos, Piñeros, Corrêa

## Conflicts of Interest

The authors declare no conflicts of interest.
